# Crystal structure and Hirshfeld surface analysis of 2,4,6-tri­amino­pyrimidine-1,3-diium dinitrate

**DOI:** 10.1107/S2056989022005333

**Published:** 2022-05-27

**Authors:** Sumra Dilshad, Emine Berrin Çınar, Arif Ali, Adeeba Ahmed, Mohd Jane Alam, Musheer Ahmad, Aiman Ahmad, Necmi Dege, Eiad Saif

**Affiliations:** a Department of Applied Chemistry, Faculty of Engineering and Technology, ZHCET, Aligarh Muslim University, Aligarh (UP), India; b Department of Physics, Faculty of Arts and Sciences, Ondokuz Mayıs University, Samsun, 55200, Turkey; c Department of Physics, Faculty of Science, Aligarh Muslim University, Aligarh, (UP), India; d Department of Computer and Electronic Engineering Technology, Sanaa Community, College, Sanaa, Yemen; Tulane University, USA

**Keywords:** crystal structure, energy framework, Hirshfeld surface, hydrogen bond, pyrimidine

## Abstract

The title compound, C_4_H_9_N_5_^2+^·2NO_3_^−^, crystallizes in the monoclinic crystal system, space group *P*2_1_/*c*. The asymmetric unit, which comprises a diprotonated tri­amino­pyrimidine dication and two nitrate anions, has an almost planar geometry with a dihedral angle of 0.92 (4)° between the mean plane of the cation and that defined by both anions. In the crystal, hydrogen-bonding inter­actions between the 2,4,6-tri­amino­pyrimidine cation and the nitrate anions lead to a one-dimensional supra­molecular network with weak anionic inter­actions forming a three-dimensional network. These inter­actions were investigated using Hirshfeld surface analysis, which indicates that the most important contributions for the packing arrangement are from O⋯H/H⋯O (53.2%), N⋯H/H⋯N (12.5%) and C⋯H/H⋯C (9.6%) inter­actions. Energy framework analysis showed that of the components of the framework energies, electrostatic repulsion (*E*_rep_) is dominant.

## Abstract 2

In the crystal, hydrogen-bonding inter­actions between the 2,4,6-tri­amino­pyrimidine cation and the nitrate anions lead to a one-dimensional supra­molecular network with weak anionic inter­actions forming a three-dimensional network. Energy framework analysis showed that of the components of the framework energies, electrostatic repulsion (*E*_rep_) is dominant.

## 1. Chemical context

**Figure 1:**
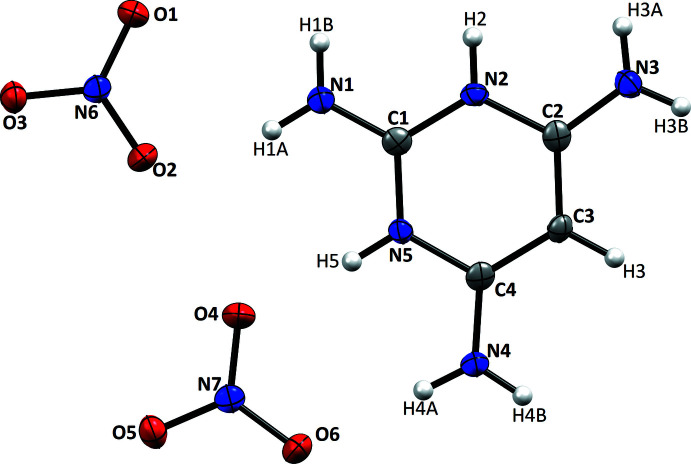
*ORTEP* diagram of the title compound with atom labeling and 50% probability ellipsoids.

Nitro­gen heterocycles and pyrimidines are examples of the most important biologically active compounds and find wide use in modern medicine (Pałasz & Cież, 2015 ▸; Takeshita *et al.*, 2006 ▸; Henderson *et al.*, 2003 ▸). Pyrimidine derivatives are used as inter­mediates for the production of various complex organic mol­ecules for the treatment of cancer and AIDS (Fawcett *et al.*, 1996 ▸). Several pyrimidine derivatives belong to the class of central nervous system depressants (Soayed *et al.*, 2015 ▸). Pyrimidine and its derivatives have great importance as they constitute a significant class of natural and non-natural products, many of which possess remarkable biological activities and clinical applications such as anti­bacterial, anti­malarial and anti­cancer agents (Sharma *et al.*, 2014 ▸). Many pyrimidine derivatives are reported to possess potential central nervous system (CNS) depressant properties and also act as calcium channel blockers (Kumar *et al.*, 2002 ▸). Pyrimido[4,5-*d*]pyrimidine-2,5-dione and 2,4-di­amino-5-(substituted)pyrimidines have been reported to have potent anti­microbial activity (Sharma *et al.*, 2004 ▸) and 2,4,6-tri­amino­pyrimidine (TAP) acts as a fast-killing and long-acting anti­malarial agent (Hameed, *et al.*, 2015 ▸). It is also known to inhibit sodium transport in the skin of frogs (Bowman *et al.*, 1978 ▸). It can be synthesized by a regioselective cyclo­addition process in high yield by reaction between two moles of cyanamide and one mole of ynamide in the presence of triflic acid as catalyst (Dubovtsev, *et al.*, 2021 ▸). Many pyrimidine derivatives display inter­esting optical and sensing properties (Achelle *et al.*, 2012 ▸, Seenan *et al.*, 2020 ▸). Methyl­pyrimidinium push–pull derivatives have been shown to be promising materials for optical data processing. Organometallic meth­yl­pyrimidinium chromophores incorporating a ruthenium fragment within the π-conjugated spacer are among the best metal–diyne NLO chromophores (Fecková, *et al.*, 2020 ▸). Herein, we report the structure of 2,4,6-tri­amino-1,3,5-tri­azine-1,3-diium dinitrate, Fig. 1 ▸, which was synthesized *via* reaction of 2,4,6-tri­amino­pyrimidine with nitric acid. 
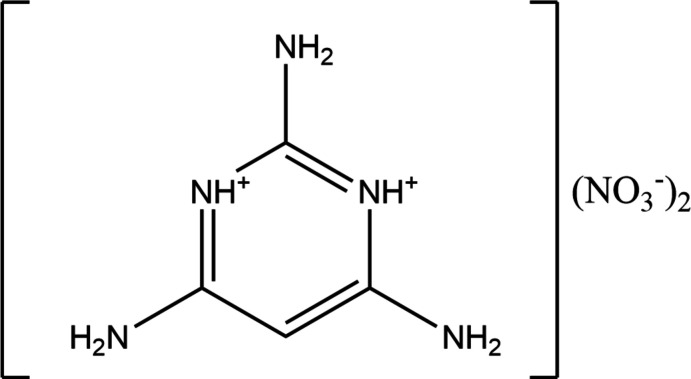


## 2. Structural commentary

In the asymmetric unit, the mean planes of the nitrate anions are inclined to one another by 5.97 (8)°. The plane of the anion containing N6 is inclined to the mean plane of the cation by 3.25 (6)° while that of the other anion is inclined by 2.84 (6)°. Thus the whole asymmetric unit lies close to a common plane (Fig.1). The ring C—N bond lengths in the cation [C1—N2 = 1.3531 (16) Å and C2—N3 = 1.3267 (16) Å] are only slightly altered from those in the corresponding conjugate base (Schwalbe *et al.*, 1982 ▸). The C—C bond lengths in the pyrimidine ring [C2—C3 = 1.3834 (18) and C3—C4 = 1.3888 (17) Å] are consistent with literature values (Ali *et al.*, 2021 ▸). The exocyclic C2—N3 and C4—N4 bond lengths [1.3267 (16) and 1.3240 (17) Å, respectively] are equivalent within experimental error but the C1—N1 bond length is markedly shorter at 1.3010 (17) Å. As it lies between the two protonated ring nitro­gen atoms, this suggests that the neighboring positive charge induces a contribution from a charge-separated quinoid form to the overall electronic structure, as has been proposed for the analogous chloride salt (Portalone & Colapietro, 2007 ▸)

## 3. Supra­molecular features

**Figure 2:**
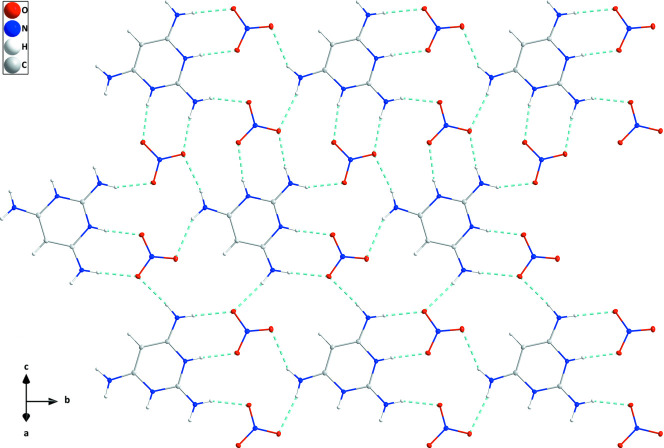
A portion of one cation/anion layer projected onto (101) with N—H⋯O hydrogen bonds depicted by dashed lines.

**Figure 3:**
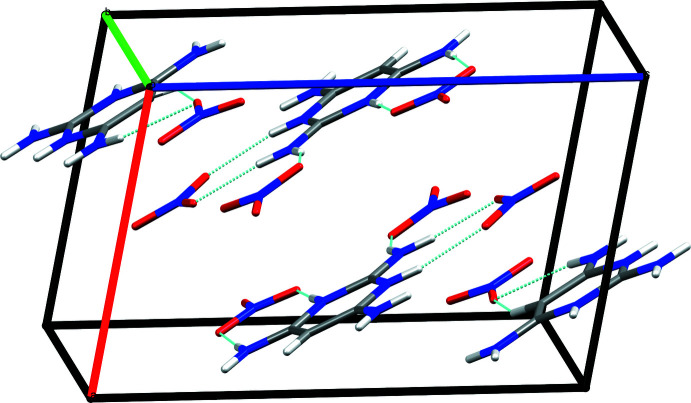
Packing view of the title compound showing the anionic–π inter­action that forms the supra­molecular structure.

In the crystal, a combination of N1—H1*A*⋯O2, N5—H5⋯O4, N4—H4*A*⋯O6, N3—H1*A*⋯O3 and N3—H3*B*⋯O5 hydrogen bonds (Table 1 ▸) leads to the formation of ribbons of alternating cations and anions extending along the *b*-axis direction. The mean planes of the ribbons are parallel to (101). Pairs of adjacent ribbons are linked by N1—H1*B*⋯O3, N2—H2⋯O1 and N3—H3*A*⋯O3 hydrogen bonds (Table 1 ▸), with these units further connected into cation/anion layers by complementary N4—H4*B*⋯O6 hydrogen bonds. The two unique nitrate ions are connected to the cation by N—H⋯O hydrogen bonds (Table 1 ▸), forming units with an 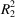 (8) graph-set motif (Fig. 2 ▸). This tight hydrogen-bonded network causes a short O2⋯O4 contact of 2.7752 (15) Å. Finally, the layers appear to be associated through N=O⋯π(ring) inter­actions N6=O3⋯*Cg*1^i^ and N7=O5⋯*Cg*1^ii^ (*Cg*1^i^ is the centroid of the pyrimidine ring at −*x* + 1, −*y* + 1, −*z* + 1; *Cg* ^ii^ is the centroid of the pyrimidine ring at −*x* + 2, −*y* + 1, −*z* + 1) with O3⋯*Cg*1^i^ = 3.1369 (11) Å, N6⋯*Cg*1^i^ = 3.4241 (12) Å, N6=O3⋯*Cg*1^i^ = 92.16 (7)°; O5⋯*Cg*1^ii^ = 3.0265 (11) Å; N7⋯*Cg*1^ii^ = 3.5176 (12) Å; N7=O5⋯*Cg*1^ii^ = 102.62 (7)° (Fig. 3 ▸).

## 4. Hirshfeld Surface Analysis

The Hirshfeld surface analysis (Spackman & Jayatilaka *et al.* 2009 ▸) was performed and the two-dimensional fingerprint plots (McKinnon *et al.*, 2007 ▸) were generated with *Crystal Explorer17* (Turner *et al.*, 2017 ▸) to qu­antify the inter­molecular contacts present within the crystal structure.

**Figure 4:**
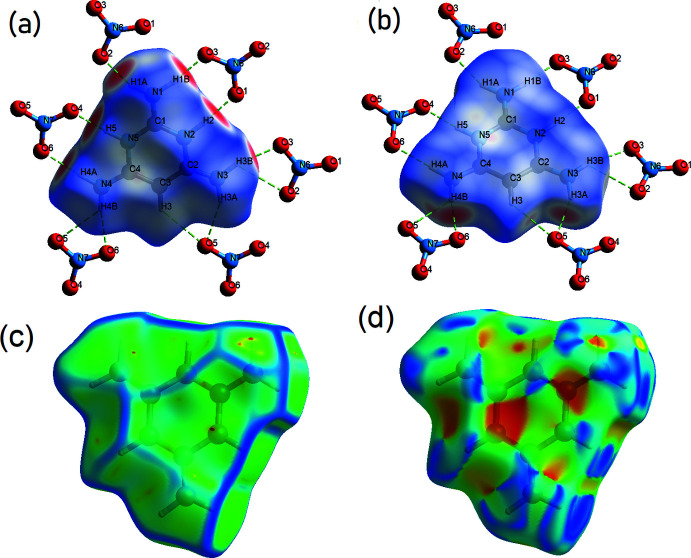
The Hirshfeld surface of the title complex mapped over (*a*) *d*_norm_ (top view), (*b*) *d*_norm_ (bottom view), (*c*) curvedness and (*d*) shape-index.

**Figure 5:**
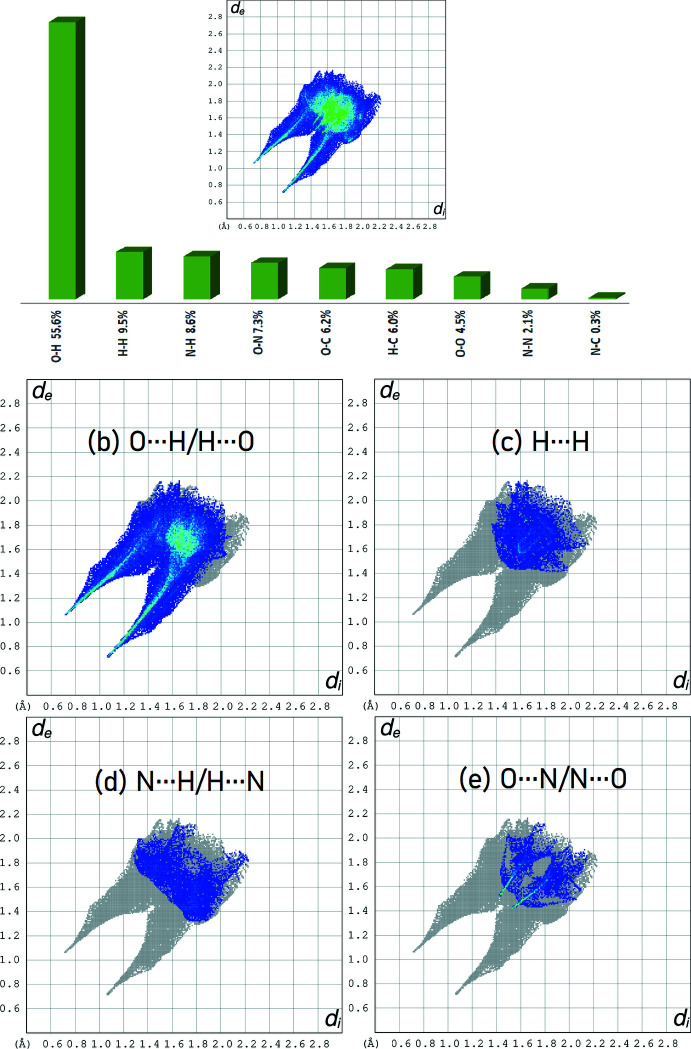
(*a*) The overall two-dimensional fingerprint plot, and those delineated into (*b*) O⋯H/H⋯O, (*c*) N⋯H/H⋯N and (*d*) C⋯H/H⋯C inter­actions.

The Hirshfeld surface is mapped over *d*_norm_ in the range −0.6823 to 0.9826 in arbitrary units with colors ranging from red (shorter distance than the sum of van der Waals radii) through white to blue (longer distance than the sum of the van der Waals radii). Top and bottom views of the surface together with curvedness, and shape-index plots are given in Fig. 4 ▸ *a*–*d*. The red spots symbolize N—H⋯O contacts and C—H⋯O inter­actions. The fingerprint plots (Fig. 5 ▸) give an insight into the overall packing characteristics of the contents of the unit cell, being plots of *d*_e_ *versus d*_i_, where *d*_i_ is the distance to the nearest atom center inter­ior to the surface, and *d*_e_ to the nearest atom exterior to the surface. These plots show that the main contributions to the overall surface involve O⋯H/H⋯O contacts at 53.2% (Fig. 5 ▸ *b*), followed by N⋯H/H⋯N contacts at 12.5% (Fig. 5 ▸ *c*) and C⋯H/H⋯C contacts at 9.6% (Fig. 5 ▸ *d*).

## 5. Synthesis and crystallization

**Figure 6:**
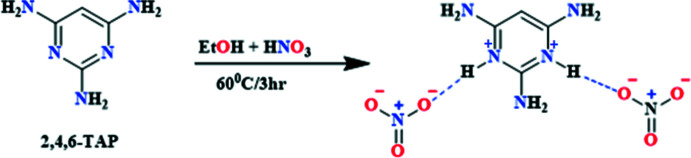
Synthesis of title compound.

To synthesize the title compound, 20 mg of 2,4,6-tri­amino­pyrimidine were dissolved in ethanol (10 mL) and the solution stirred for 3 h. A mixture of ethanol (5 mL) and nitric acid (0.5 mL) was taken in a separate round-bottom flask and stirred for 3 h at 333 K. Afterwards, the 2,4,6-tri­amino­pyrimidine solution was added dropwise to the above mixture. The reaction was continued for 4 h at the same temperature. After completion of the reaction, a pale-yellow solution was obtained, which was filtered and kept for slow evaporation at room temperature. After 15 days, pale-yellow crystals were obtained that were suitable for data collection (Fig. 6 ▸).

## 6. Inter­action energy calculations

**Figure 7:**
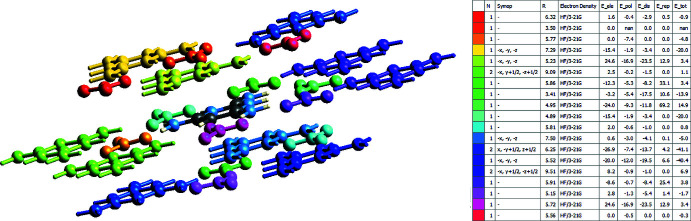
Inter­action energies for the title compound were calculated with the HF/3–21 G model.

**Figure 8:**
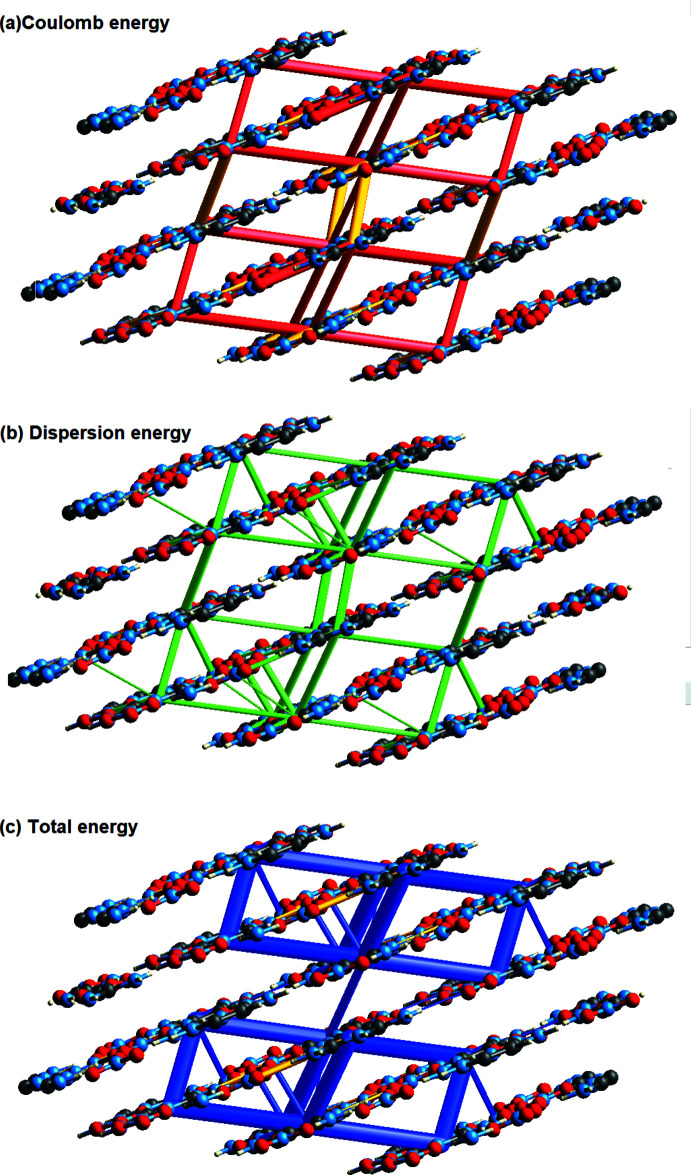
Energy frameworks for a 2×2×2 supercell viewed down the crystallographic *b* axis for the threefold inter­penetrated crystal structure. The red-colored frame shows the Coulombic energy, green shows dispersion, and blue shows total energy.

The inter­action energies for the title compound (Fig. 7 ▸). were computed using the HF/3-21G quantum level of theory, which is available in *CrystalExplorer 17.5*. Electrostatic (*E*_ele_), polarization (*E*_pol_), dispersion (*E*_disp_), and exchange-repulsion (*E*_rep_) are the four energy variables that make up the total inter­molecular inter­action energy (*E*_tot_). Cylinder-shaped energy frameworks represent the relative strengths of inter­action energies in individual directions and give the topologies of pair-wise inter­molecular inter­action energies within the crystal (Mackenzie *et al.*, 2017 ▸). The energies between mol­ecular pairs are represented as cylinders connecting the centroids of pairs of mol­ecules, with the cylinder radius equal to the amount of inter­action energy between the mol­ecules (Wu *et al.*, 2020 ▸). The dark-blue-colored mol­ecule at symmetry position (*x*, −*y* + 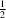 , *z* + 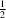 ) located 6.25 Å from the centroid of the selected mol­ecule has the highest total inter­action energy of −40.1 kJ mol^−1^, as shown in Fig.7. The net inter­action energies for the title compound are *E*_ele_ = −58.9 kJ mol^−1^, *E*_pol_ = −92.0 kJ mol^−1^, *E*_dis_ = −148.8 kJ mol^−1^, *E*_rep_ = 176.9 kJ mol^−1^, with a total inter­action energy *E*_tot_ of −110.4 kJ mol^−1^ (Fig. 8 ▸). Clearly, *E*_rep_ is the major inter­action energy in the title compound.

## 7. Database survey

A search of the Cambridge Structural Database (CSD, Version 5.43, update of March 2022; Groom *et al.*, 2016 ▸) for the tri­amino­pyrimidine dication gave 24 hits of which 16 were for the FPO_3_^2−^ salt studied at a variety of temperatures (GESWAF–GESWAF15; Matulková *et al.*, 2017 ▸) but no structure containing nitrate anions was found. The remaining structures contain arene­sulfonate (TEYTEZ, TEYTID and TEYXIH; Karak *et al.*, 2018 ▸), various polycarboxyl­ate (VEXQEX, VEXZUW and VEYBEJ; Xing, *et al.*, 2017 ▸), chloride (GIMROK; Portalone & Colapietro, 2007 ▸) and [Cu_2_Cl_8_]^4−^ (GOHDOY; Voronina *et al.*, 2012 ▸) anions. Most of the discussions of these structures are concerned more with their supra­molecular structures than the detailed geometry of the cation but, as noted in Section 3, some details similar to those in the present work are seen in the structure of the chloride salt.

## 8. Refinement

Crystal data, data collection and structure refinement details are summarized in Table 2 ▸. All H atoms were originally found in difference maps. Thy were positioned geometrically (N—H = 0.86 Å, C—H = 0.93 Å) and refined as riding with *U*_iso_(H) = 1.2*U*_eq_(C,N).

## Supplementary Material

Crystal structure: contains datablock(s) I. DOI: 10.1107/S2056989022005333/mw2185sup1.cif

Structure factors: contains datablock(s) I. DOI: 10.1107/S2056989022005333/mw2185Isup2.hkl

CCDC reference: 2173730

Additional supporting information:  crystallographic information; 3D view; checkCIF report

## Figures and Tables

**Table 1:** Hydrogen-bond geometry (Å, °)

*D*—H⋯*A*	*D*—H	H⋯*A*	*D*⋯*A*	*D*—H⋯*A*
N5—H5⋯O4	0.86	1.88	2.7321 (15)	174
N2—H2⋯O1^i^	0.86	1.98	2.8319 (15)	169
N4—H4*A*⋯O6	0.86	2.08	2.9428 (15)	177
N4—H4*B*⋯O5^ii^	0.86	2.43	3.0706 (16)	131
N4—H4*B*⋯O6^ii^	0.86	2.11	2.9593 (15)	172
N1—H1*A*⋯O2	0.86	1.97	2.7986 (15)	160
N1—H1*B*⋯O3^i^	0.86	1.94	2.7912 (15)	172
N3—H3*A*⋯O3^iii^	0.86	2.16	3.0018 (14)	167
N3—H3*A*⋯O2^iii^	0.86	2.54	2.9770 (15)	113
N3—H3*B*⋯O5^iii^	0.86	2.26	3.0619 (15)	156
C3—H3⋯O5^iii^	0.93	2.56	3.3134 (16)	139

Symmetry codes: (i) 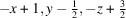 ; (ii) 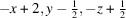 ; (iii) 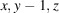 .

**Table 2:** Experimental details

Crystal data	
Chemical formula	C_4_H_9_N_5_^2+^·2NO_3_^−^
*M*_r_	251.18
Crystal system, space group	Monoclinic, *P*2_1_/*c*
Temperature (K)	276
*a*, *b*, *c* (Å)	7.8650 (5), 9.9173 (6), 12.2291 (7)
β (°)	100.836 (2)
*V* (Å^3^)	936.86 (10)
*Z*	4
Radiation type	Mo *K*α
μ (mm^−1^)	0.16
Crystal size (mm)	0.37 × 0.27 × 0.14

Data collection	
Diffractometer	Bruker APEXII CCD
No. of measured, independent and observed [*I* > 2σ(*I*)] reflections	13469, 2312, 1993
*R*_int_	0.070
(sin θ/λ)_max_ (Å^−1^)	0.668

Refinement	
*R*[*F* ^2^ > 2σ(*F* ^2^)], *wR*(*F* ^2^), *S*	0.040, 0.110, 1.07
No. of reflections	2312
No. of parameters	154
H-atom treatment	H-atom parameters constrained
Δρ_max_, Δρ_min_ (e Å^−3^)	0.51, −0.30

Computer programs: *X-AREA* and *X-RED32* (Stoe & Cie, 2002 ▸), *SHELXT2018/3* (Sheldrick, 2015*a* ▸), *SHELXL2018/3* (Sheldrick, 2015*b* ▸), *OLEX2* (Dolomanov *et al.*, 2009 ▸), *Mercury* (Macrae *et al.*, 2020 ▸), *WinGX* (Farrugia, 2012 ▸), *PLATON* (Spek, 2020 ▸) and *publCIF* (Westrip, 2010 ▸).

## supplementary crystallographic information

### Crystal data

**Table:**

C_4_H_9_N_5_^2+^·2NO_3_^−^	*F*(000) = 520
*M**_r_* = 251.18	*D*_x_ = 1.781 Mg m^−^^3^
Monoclinic, *P*2_1_/*c*	Mo *K*α radiation, λ = 0.71073 Å
*a* = 7.8650 (5) Å	Cell parameters from 8448 reflections
*b* = 9.9173 (6) Å	θ = 2.3–25.6°
*c* = 12.2291 (7) Å	µ = 0.16 mm^−^^1^
β = 100.836 (2)°	*T* = 276 K
*V* = 936.86 (10) Å^3^	Needle, colourless
*Z* = 4	0.37 × 0.27 × 0.14 mm

### Data collection

**Table:**

Bruker APEXII CCD diffractometer	*R*_int_ = 0.070
φ and ω scans	θ_max_ = 28.3°, θ_min_ = 2.6°
13469 measured reflections	*h* = −10→10
2312 independent reflections	*k* = −13→13
1993 reflections with *I* > 2σ(*I*)	*l* = −16→16

### Refinement

**Table:**

Refinement on *F*^2^	0 restraints
Least-squares matrix: full	Hydrogen site location: inferred from neighbouring sites
*R*[*F*^2^ > 2σ(*F*^2^)] = 0.040	H-atom parameters constrained
*wR*(*F*^2^) = 0.110	*w* = 1/[σ^2^(*F*_o_^2^) + (0.0497*P*)^2^ + 0.4576*P*] where *P* = (*F*_o_^2^ + 2*F*_c_^2^)/3
*S* = 1.07	(Δ/σ)_max_ < 0.001
2312 reflections	Δρ_max_ = 0.51 e Å^−^^3^
154 parameters	Δρ_min_ = −0.30 e Å^−^^3^

### Special details

**Table:**

Geometry. All esds (except the esd in the dihedral angle between two l.s. planes) are estimated using the full covariance matrix. The cell esds are taken into account individually in the estimation of esds in distances, angles and torsion angles; correlations between esds in cell parameters are only used when they are defined by crystal symmetry. An approximate (isotropic) treatment of cell esds is used for estimating esds involving l.s. planes.

### Fractional atomic coordinates and isotropic or equivalent isotropic displacement parameters (Å^2^)

**Table:**

	*x*	*y*	*z*	*U*_iso_*/*U*_eq_	
O3	0.54263 (12)	0.80420 (9)	0.71416 (8)	0.0141 (2)	
O5	0.88490 (13)	0.76472 (9)	0.36706 (8)	0.0148 (2)	
O1	0.50750 (13)	0.59279 (10)	0.75438 (8)	0.0168 (2)	
O6	0.93192 (13)	0.56691 (10)	0.30278 (8)	0.0173 (2)	
O4	0.80491 (14)	0.58662 (10)	0.44532 (8)	0.0207 (2)	
O2	0.64974 (14)	0.64786 (10)	0.62512 (9)	0.0205 (2)	
N5	0.77821 (13)	0.31443 (11)	0.47214 (9)	0.0099 (2)	
H5	0.787384	0.398958	0.458644	0.012*	
N2	0.68341 (13)	0.14353 (11)	0.57534 (9)	0.0105 (2)	
H2	0.634287	0.118719	0.629218	0.013*	
N4	0.92308 (15)	0.27225 (11)	0.32949 (9)	0.0128 (2)	
H4A	0.929637	0.358122	0.321423	0.015*	
H4B	0.967123	0.218902	0.286854	0.015*	
N6	0.56691 (14)	0.68070 (11)	0.69851 (9)	0.0115 (2)	
N7	0.87310 (14)	0.63926 (11)	0.37153 (9)	0.0116 (2)	
N1	0.63901 (14)	0.36592 (11)	0.61695 (9)	0.0130 (2)	
H1A	0.649848	0.450490	0.604304	0.016*	
H1B	0.588658	0.340225	0.670025	0.016*	
N3	0.71841 (14)	−0.08175 (11)	0.53700 (9)	0.0131 (2)	
H3A	0.666228	−0.100504	0.590919	0.016*	
H3B	0.754039	−0.145693	0.499473	0.016*	
C1	0.69879 (15)	0.27685 (13)	0.55581 (10)	0.0103 (3)	
C4	0.84517 (15)	0.22217 (13)	0.40753 (10)	0.0101 (3)	
C3	0.82632 (16)	0.08538 (12)	0.42648 (10)	0.0103 (3)	
H3	0.868341	0.021484	0.382632	0.012*	
C2	0.74394 (15)	0.04582 (13)	0.51170 (10)	0.0106 (2)	

### Atomic displacement parameters (Å^2^)

**Table:**

	*U*^11^	*U*^22^	*U*^33^	*U*^12^	*U*^13^	*U*^23^
O3	0.0184 (5)	0.0101 (4)	0.0138 (5)	0.0018 (3)	0.0028 (4)	−0.0004 (3)
O5	0.0195 (5)	0.0105 (5)	0.0130 (5)	−0.0005 (4)	−0.0006 (4)	0.0002 (3)
O1	0.0244 (5)	0.0126 (5)	0.0153 (5)	−0.0007 (4)	0.0088 (4)	0.0021 (3)
O6	0.0254 (5)	0.0144 (5)	0.0140 (5)	0.0018 (4)	0.0088 (4)	−0.0018 (4)
O4	0.0344 (6)	0.0149 (5)	0.0166 (5)	−0.0011 (4)	0.0142 (4)	0.0018 (4)
O2	0.0328 (6)	0.0141 (5)	0.0189 (5)	0.0008 (4)	0.0163 (4)	−0.0014 (4)
N5	0.0140 (5)	0.0081 (5)	0.0071 (5)	−0.0002 (4)	0.0005 (4)	0.0004 (4)
N2	0.0133 (5)	0.0113 (5)	0.0068 (5)	−0.0011 (4)	0.0018 (4)	0.0008 (4)
N4	0.0187 (5)	0.0116 (5)	0.0087 (5)	0.0005 (4)	0.0041 (4)	0.0004 (4)
N6	0.0140 (5)	0.0119 (5)	0.0077 (5)	0.0004 (4)	−0.0001 (4)	−0.0004 (4)
N7	0.0126 (5)	0.0133 (5)	0.0078 (5)	0.0003 (4)	−0.0014 (4)	0.0004 (4)
N1	0.0181 (5)	0.0106 (5)	0.0106 (5)	−0.0001 (4)	0.0033 (4)	0.0000 (4)
N3	0.0174 (5)	0.0101 (5)	0.0115 (5)	−0.0005 (4)	0.0020 (4)	0.0011 (4)
C1	0.0095 (5)	0.0127 (6)	0.0071 (5)	−0.0003 (4)	−0.0026 (4)	0.0002 (4)
C4	0.0103 (5)	0.0125 (6)	0.0056 (5)	0.0000 (4)	−0.0032 (4)	−0.0006 (4)
C3	0.0122 (5)	0.0107 (6)	0.0072 (5)	0.0009 (4)	−0.0006 (4)	−0.0015 (4)
C2	0.0095 (5)	0.0118 (6)	0.0082 (5)	0.0000 (4)	−0.0040 (4)	−0.0008 (4)

### Geometric parameters (Å, º)

**Table:**

O3—N6	1.2597 (14)	N4—C4	1.3240 (17)
O5—N7	1.2496 (14)	N4—H4A	0.8600
O1—N6	1.2511 (15)	N4—H4B	0.8600
O6—N7	1.2577 (15)	N1—C1	1.3010 (17)
O4—N7	1.2476 (15)	N1—H1A	0.8600
O2—N6	1.2473 (15)	N1—H1B	0.8600
N5—C1	1.3477 (16)	N3—C2	1.3267 (16)
N5—C4	1.3767 (16)	N3—H3A	0.8600
N5—H5	0.8600	N3—H3B	0.8600
N2—C1	1.3531 (16)	C4—C3	1.3888 (17)
N2—C2	1.3822 (16)	C3—C2	1.3834 (18)
N2—H2	0.8600	C3—H3	0.9300
			
C1—N5—C4	122.26 (11)	H1A—N1—H1B	120.0
C1—N5—H5	118.9	C2—N3—H3A	120.0
C4—N5—H5	118.9	C2—N3—H3B	120.0
C1—N2—C2	122.27 (11)	H3A—N3—H3B	120.0
C1—N2—H2	118.9	N1—C1—N5	121.18 (12)
C2—N2—H2	118.9	N1—C1—N2	120.53 (12)
C4—N4—H4A	120.0	N5—C1—N2	118.28 (11)
C4—N4—H4B	120.0	N4—C4—N5	116.31 (11)
H4A—N4—H4B	120.0	N4—C4—C3	124.41 (12)
O2—N6—O1	120.68 (11)	N5—C4—C3	119.28 (11)
O2—N6—O3	118.54 (11)	C2—C3—C4	118.85 (12)
O1—N6—O3	120.78 (11)	C2—C3—H3	120.6
O4—N7—O5	119.59 (11)	C4—C3—H3	120.6
O4—N7—O6	120.46 (11)	N3—C2—N2	117.00 (11)
O5—N7—O6	119.94 (11)	N3—C2—C3	123.98 (12)
C1—N1—H1A	120.0	N2—C2—C3	119.02 (11)
C1—N1—H1B	120.0		
			
C4—N5—C1—N1	178.85 (11)	N4—C4—C3—C2	179.23 (12)
C4—N5—C1—N2	−0.76 (17)	N5—C4—C3—C2	−1.46 (17)
C2—N2—C1—N1	179.46 (11)	C1—N2—C2—N3	−178.59 (10)
C2—N2—C1—N5	−0.93 (17)	C1—N2—C2—C3	1.36 (17)
C1—N5—C4—N4	−178.68 (11)	C4—C3—C2—N3	179.81 (11)
C1—N5—C4—C3	1.96 (17)	C4—C3—C2—N2	−0.13 (17)

### Hydrogen-bond geometry (Å, º)

**Table:**

*D*—H···*A*	*D*—H	H···*A*	*D*···*A*	*D*—H···*A*
N5—H5···O4	0.86	1.88	2.7321 (15)	174
N2—H2···O1^i^	0.86	1.98	2.8319 (15)	169
N4—H4*A*···O6	0.86	2.08	2.9428 (15)	177
N4—H4*B*···O5^ii^	0.86	2.43	3.0706 (16)	131
N4—H4*B*···O6^ii^	0.86	2.11	2.9593 (15)	172
N4—H4*B*···N7^ii^	0.86	2.62	3.4400 (16)	160
N1—H1*A*···O2	0.86	1.97	2.7986 (15)	160
N1—H1*A*···N6	0.86	2.69	3.3577 (16)	135
N1—H1*B*···O3^i^	0.86	1.94	2.7912 (15)	172
N3—H3*A*···O3^iii^	0.86	2.16	3.0018 (14)	167
N3—H3*A*···O2^iii^	0.86	2.54	2.9770 (15)	113
N3—H3*B*···O5^iii^	0.86	2.26	3.0619 (15)	156
C3—H3···O5^iii^	0.93	2.56	3.3134 (16)	139

Symmetry codes: (i) −*x*+1, *y*−1/2, −*z*+3/2; (ii) −*x*+2, *y*−1/2, −*z*+1/2; (iii) *x*, *y*−1, *z*.
